# Supplementary material for: Coexistence of electron whispering-gallery modes and atomic collapse states in graphene/WSe2 heterostructure quantum dots
Source: Nat Commun. 2022 Mar 24;13:1597. doi: 10.1038/s41467-022-29251-2 (PMC8948210; doi:10.1038/s41467-022-29251-2)
Supplement: Supplementary file 1 — Supplementary Information [file 41467_2022_29251_MOESM1_ESM.pdf]

SUPPLEMENTARY INFORMATION

Coexistence of electron whispering-gallery modes and atomic collapse states in graphene/WSe_2 heterostructure quantum dots

Qi Zheng[1,§], Yu-Chen Zhuang[2,§], Qing-Feng Sun[2,3,4,†], Lin He[1,†]

Affiliations:
[1] Center for Advanced Quantum Studies, Department of Physics, Beijing Normal University, Beijing, 100875, People's Republic of China
[2] International Center for Quantum Materials, School of Physics, Peking University, Beijing, 100871, China
[3] Collaborative Innovation Center of Quantum Matter, Beijing 100871, China
[4] Beijing Academy of Quantum Information Sciences, West Bld. #3, No. 10 Xibeiwang East Road, Haidian District, Beijing 100193, China

[§]These authors contributed equally to this work.
[†]Correspondence and requests for materials should be addressed to Qing-Feng Sun (email: sunqf@pku.edu.cn) and Lin He (e-mail: helin@bnu.edu.cn).

CONTENTS

1. **Discussion on the origin of the WSe_2 quantum dots**
2. **STM and FFT images of a typical GQD with different tip bias**
3. **WGMs confinement in the GQDs**
4. **The dangling bonds of WSe_2 induce confining potential in graphene**
5. **Theoretical model**
6. **Theoretical model in the presence of magnetic fields**
7. **Numerical tight-binding approach**
8. **The d*I*/d*V* spectra and simulated LDOS at different GQDs**
9. **Spatial distribution of ACSs in the GQD**
10. **Simulated LDOS of quasibound states in the GQD (r_0 = 9 nm) as a function of β and energy**
11. **Screened electrostatic potential and asymmetry of electron and hole branches**
12. **The others radially d*I*/d*V* spectroscopic maps under a series of magnetic fields**
13. **The d*I*/d*V* spectra and simulated LDOS at $B = 10$ T**
14. **The corresponding d*I*/d*V* spectra to experimental data in Fig. 4 in main text and calculated LDOS map with different energy and magnetic field scale**
15. **The calculated LDOS map for Gaussian potential**
16. **The calculated LDOS map for parabolic potential**

1. Discussion on the origin of the WSe_2 quantum dots

Based on atomic force microscopy (AFM) analysis of the freshly mechanical exfoliated WSe_2 surface and after transferring the graphene monolayer (Fig. S1), it is reasonably to assume that the WSe_2 quantum dots (QDs) are naturally generated during the process of mechanical exfoliated. Around these WSe_2 QDs, a few WSe_2 anti-dots usually can be observed in the STM images (Fig. S2a). Such a result indicates that nanoscaled WSe_2 flakes are pulled off the substrate and then fall down to the substrate to form the WSe_2 QDs in the process of mechanical exfoliation.

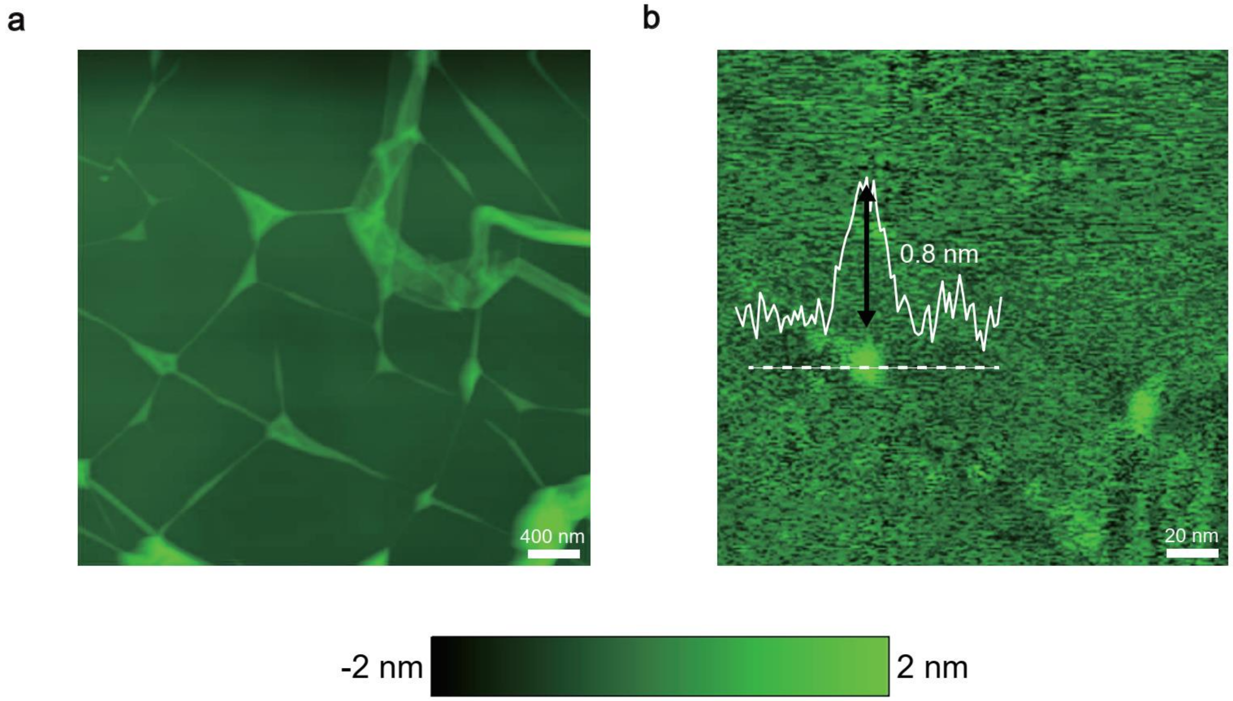

Fig. S1. a, Large-area AFM topograph of graphene/WSe_2 heterostructure. The large and flat area can be observed in addition to some bubbles. **b**, Zoom-in image of **a**. The WSe_2 QD with around 0.8 nm in height (WSe_2 monolayer).

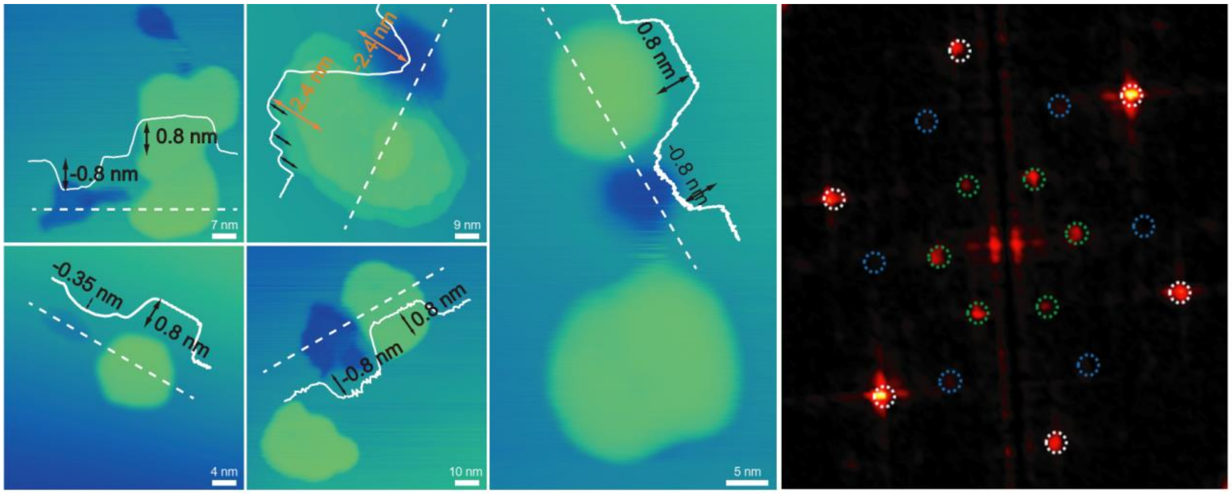

Fig. S2. a, The STM images of GQDs ($V = -0.8$ V, $I = 100$ pA) and height profiles (solid white line) along the white dotted line (the thickness of monolayer WSe_2 is about 0.8 nm). A few anti-dots (-0.8 nm) usually can be observed near the GQDs. **b**, FFT of the GQD in Fig. 1**b**. the bright spots in the white dotted circles represent the reciprocal lattice of graphene, the bright spots in the blue dotted circles represent the reciprocal lattice of WSe_2, and the bright spots in the green dotted circles represent moiré structures from the graphene/WSe_2 heterostructure.

2. STM and FFT images of a typical GQD with different tip bias

In Fig. 1c of main text, there is one-dimensional (1D) stripe-like feature on the GQD, which arises from the moiré pattern formed by graphene/WSe_2 heterostructure. When we set the tip bias as $V = 400$ mV, the STM image shows 1D stripe-like feature on the GQD, as shown in the Fig. S3a and b. The direction of the stripe-like structures coincides with the one of the vectors of the moiré structures marked by yellow arrow. As shown in Fig. S3c, the FFT image inside the GQD (Fig. S3b) indicates moiré structure with obvious anisotropy: the moiré reciprocal vector marked by the yellow arrow presents a stronger signal. When we set the tip bias $V = -600$ mV, we cannot

observe any obvious stripe-like structures (Fig. S3d and e). The signals of the FFT in the three directions of moiré structure are basically the same (Fig. S3f).

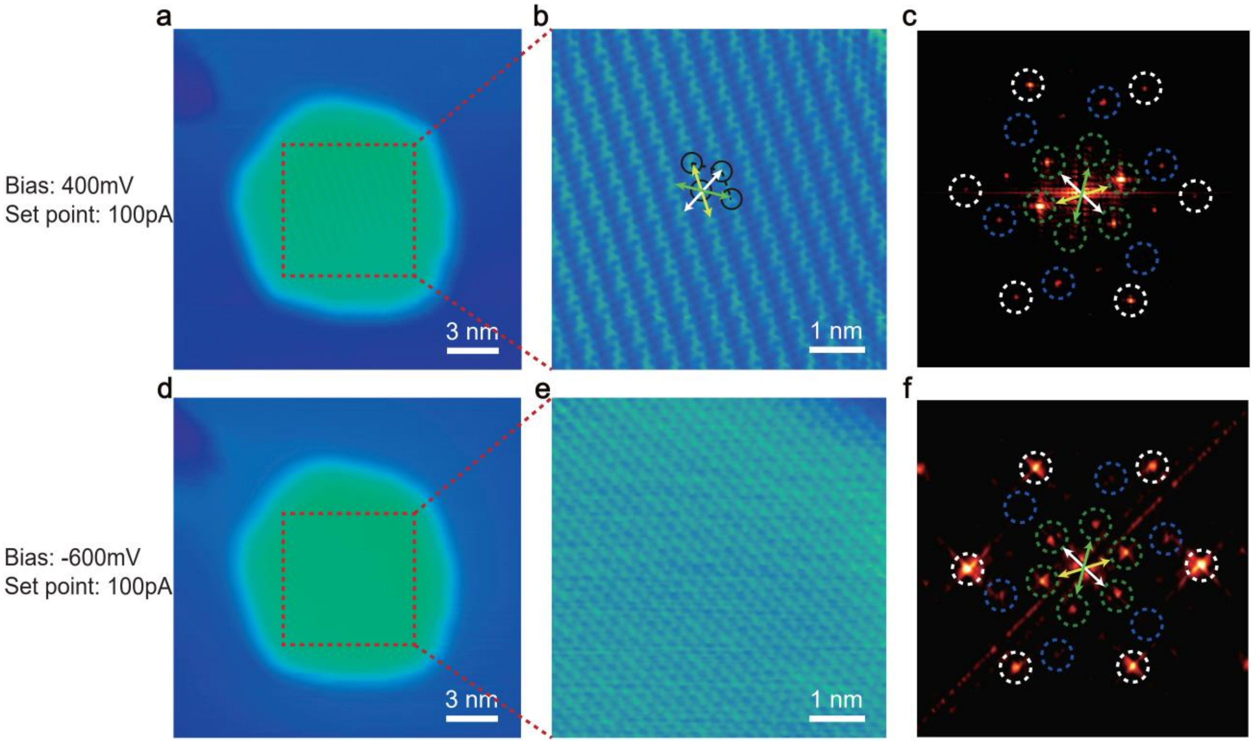

Fig. S3. The STM and corresponding FFT images of a typical graphene/WSe_2 heterostructure QD with different tip bias. **a** and **d**, The STM images with different tip bias, parameters: bias V = 400 mV, set point I = 100 pA in **a**; bias V = -600 mV, set point I = 100 pA in **d**. We can see one-dimensional stripe-like feature on the GQD in **a**. **b** and **e**, The zoom-in image of the area in red dashed squares from panel **a** and **d**. The black circles show the moiré pattern formed by graphene/WSe_2 heterostructure as shown in **b**. The three colored arrows indicate vectors of the moiré structures. **c** and **f**, FFT of the GQD in **b** and **e**. the bright spots in the white dotted circles represent the reciprocal lattice of graphene, the bright spots in the blue dotted circles represent the reciprocal lattice of WSe_2, and the bright spots in the green dotted circles represent moiré structures

from the graphene/WSe_2 heterostructure. The three colored arrows in the center of the figure indicate the direction of the three inverted vectors of the moiré structure.

3. WGMs confinement in the GQDs

As shown in Fig. 1f and Fig. S4, the quasibound state marked by N1 displays strong intensity near the center of the GQD, and higher quasibound states (marked by N2~N4) exhibits maxima which are progressively closer to the edge of the GQD. These equally spaced quasibound states confined in the GQD, which can be described by the whispering-gallery modes (WGMs) confinement.

Based on WGMs confinement of massless Dirac fermions in graphene, the quasibound states spacing is related to the radius of the GQDs by $\Delta E = \alpha\hbar v_F / R_{eff}$, where α is a dimensionless constant of order unity, $\hbar$ is Planck's constant divided by 2π, $v_F \approx 9.0\times10^5$ m/s is the Fermi velocity of the graphene monolayer, and R_{eff} denotes the effective radius of the GQDs (*1*-*3*). Since the QDs are not standard circle shapes, we define the effective radius by $R_{eff} = \sqrt{A/\pi}$, where A is the area of the GQD measured from STM images. Four representative STS spectra recorded on the GQDs with different sizes are shown in Fig. S5c, which indicates that the average energy spacing of these quasibound states decreases with the increase of the effective radius R_{eff} . In Fig. S5d, the average energy spacing of the quasibound states (ΔE) have summarized as a function of the inverse effective radius ($1/R_{eff}$) of the GQDs. The result agrees well with the confinement of the massless Dirac fermions in the GQDs, which can be described well by $\Delta E = \alpha\hbar v_F / R_{eff}$ with $\alpha \approx 1$.

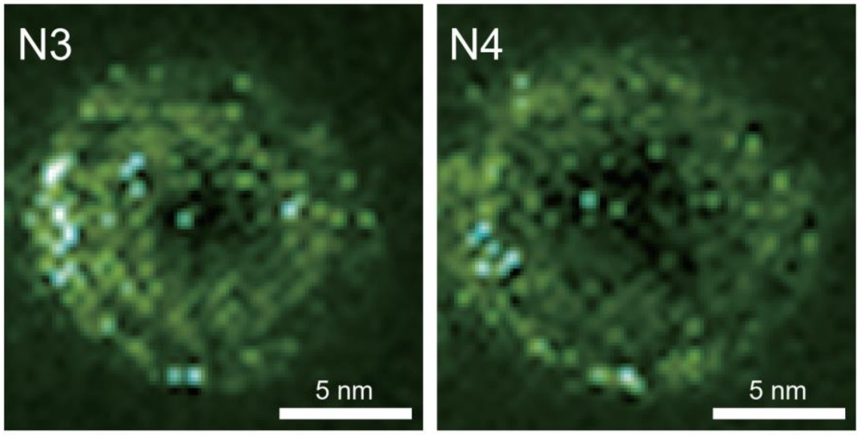

Fig. S4. The d*I*/d*V* maps (N3 and N4 marked in the Fig. 1**e**) of the GQD (Fig. 1**b**). The maps exhibit maxima progressively closer to the edge of the GQD, which can be described by the WGM confinement.

4. The dangling bonds of WSe_2 induce confining potential in graphene

We summarized the measured potential difference (ΔU, which is obtained according to the Dirac point from the d*I*/d*V* spectra) between inside and outside the graphene quantum dots (GQDs) as a function of the ratio (η) of the number of boundary atoms to the inner atoms. The ΔU roughly increases linear with the ratio η (Fig. S5a). Here, the ratio is estimated according by $\eta = \frac{l \times a}{\pi \times R_{eff}^2}$, where l and R_{eff} are the circumference and effective radius of the quasi-circle WSe_2 QDs, a = 0.353 nm denotes the WSe_2 lattice constant (*4-5*), as shown in Fig. S5a for schematic diagram of a WSe_2 QD. The linear dependence between the ΔU and the ratio η is quite reasonable. There will be many danging bonds due to the unsaturated atoms at the boundary of the WSe_2 QD. These dangling bonds (*5-6*) can significantly change the electronic structure and work function of the WSe_2 island, resulting in a circular electrostatic potential of the graphene covering it. As the size of the island increases, the ratio of the number of boundary atoms to the inner atoms decreases,

which will reduce the impact on the work function of the QD, and ultimately lead to a reduction in the potential difference between inside and outside the GQDs. This result has significant effects in introducing electrostatic potential into graphene systems.

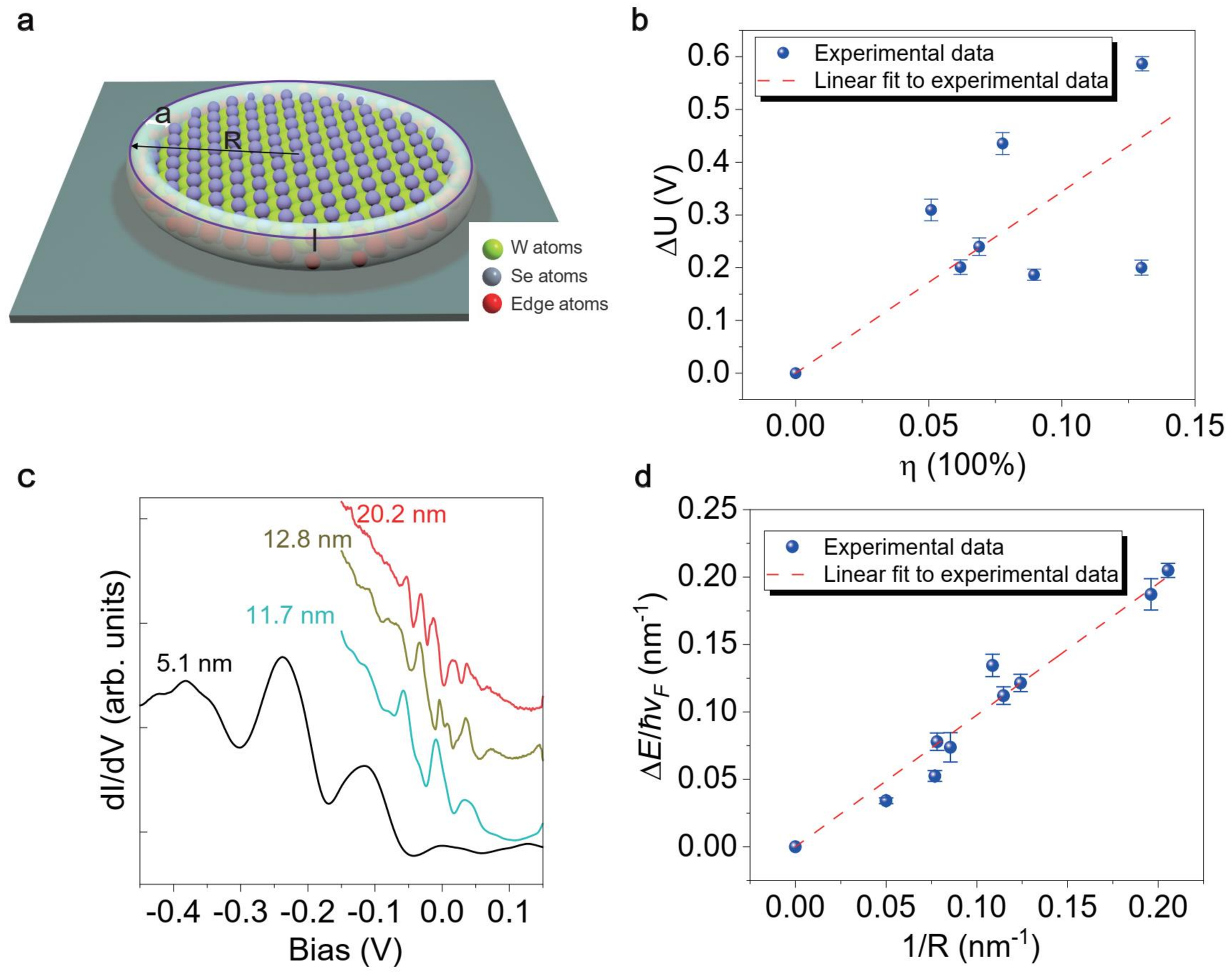

Fig. S5. The effect of island size on the confining potential and quasibound states. **a**, Schematics of a WSe_2 QD, $a = 0.353$ nm denotes the WSe_2 lattice constant, R indicates radius of WSe_2 island, l is the circumference of the quasi-circle WSe_2 island. **b**, Plot of average potential difference inside and outside the GQDs as a function of the ratio ($\eta = \frac{l \times a}{\pi \times R_{eff}^2}$) of the number of boundary atoms to the inner atoms. **c**, dI/dV spectrum for GQDs with different effective radius. **d**, Plot of average

level spacing for quasibound states as a function of inverse effective radius for GQDs, which can be described well by $\Delta E = \alpha\hbar v_F / R$ with $\alpha \approx 1$.

5. Theoretical model

The tight-binding Hamiltonian for graphene in the presence of the quantum dot is given by (*7*)

$$H = \sum_{\langle ij\rangle}(t_{ij}a_i^{\dagger}b_j + H.c) + \sum_i (V(\mathbf{r}_i^A) + \varepsilon_i^A)a_i^{\dagger}a_i + \sum_i (V(\mathbf{r}_i^B) + \varepsilon_i^B)b_i^{\dagger}b_i .$$

Here t_{ij} is the hopping energy between site *i* and site *j* in the graphene and is directly related to the Fermi velocity v_F by the relation $\hbar v_F = \frac{3}{2}t_{ij}a_{cc}$ where $\hbar$ is reduced Planck constant and a_{cc} is the lattice constant (a_{cc} = 0.142 nm). Unless otherwise stated, the Fermi velocity $v_F \approx 9\times10^5\,\mathrm{m/s}$ and $t_{ij} = -2.8eV$ are used. In this work, we only consider the nearest hopping energy and higher hopping terms do not affect our results. $a_i(a_i^{\dagger})$ and $b_i(b_i^{\dagger})$ denote the annihilation (creation) operators for A and B sublattice at site *i*. $\mathbf{r}_i^{\mathrm{A,B}}$ denotes the position relative to the GQD. $\varepsilon_i^{\mathrm{A,B}}$ and $V(\mathbf{r}_i^{\mathrm{A,B}})$ are the on-site energy and potential of atoms at each sublattice. For a GQD, we model its potential as a Coulomb form with a finite cut-off length r_0 which can be described as (*8*)

$$V_\beta(r) = \begin{cases} \hbar v_F \dfrac{\beta}{r_0}, & r \le r_0 \\ \hbar v_F \dfrac{\beta}{r}, & r > r_0 \end{cases}$$

Different from the artificial nucleus (*9*) or charge vacancy (*10*) studied in the previous experiments, the potential originates from GQD is Coulomb repulsive due to the repulsion between electrons. We argue that repulsive potential can lead to 'hole atomic collapse states' in which their energies are always higher than the bulk Dirac point and extend towards positive infinity. Its physics content

is parallel to the ACSs in view of particle-hole symmetry in the Dirac cone, so that we don't distinguish them in the following. $\beta = \frac{Ze^2}{\kappa\hbar v_F}$ is the dimensionless coupling constant (κ is relative permittivity). The cut-off length r_0 should be consistent with the size of the GQDs. They are commonly a few nanometers in size, ten times larger than the artificial nucleus. The value of β can be inferred by analyzing the difference between the inner and outer Dirac points in the experiment. We estimate that β is roughly between 2 to 5 in our experiment. It is much higher than the critical value $\beta_c = \frac{1}{2}$ for the appearance of ACSs (*8*,*11-13*). The values of $\varepsilon_i^{\mathrm{A,B}}$ can be also deduced from the measured positions of energy states experimentally.

6. Theoretical model in the presence of magnetic fields

The magnetic field effect can be also included in our simulation. In the presence of a uniform perpendicular magnetic field **B**, the hopping energy is replaced by the Peierls substitution $t_{ij} \to t_{ij} e^{i2\pi\Phi_{ij}}$, where $\Phi_{ij} = \frac{1}{\Phi_0}\int_{\vec{r}_i}^{\vec{r}_j} \vec{A}\cdot d\vec{l}$ is the Peierls phase, $\Phi_0 = \frac{h}{e}$ is the magnetic quantum flux. Under strong magnetic field, Landau levels can arise as

$$E_N = \pm \frac{\hbar v_F}{l_B}\sqrt{2|N|} \tag{1}$$

where $l_B = \sqrt{\hbar/(eB)}$ is the magnetic length, N = 0, ±1, ±2… is the LLs index, ± indicate the electron/hole branch with N > 0/N <0. When the system is uniform, each Landau level is degenerate consisting of an infinite number of orbital states with orbital number $m \geq -|N|$ (*7*). The existence of GQD could break the translation symmetry and lift the Landau level degeneracy into m-dependent sublevels E_{Nm} (*8*).

7. Numerical tight-binding approach

In the computation detail, we build a large hexagonal flake graphene system with all armchair edges (to avoid zigzag edge states with low energy). The width of hexagon edge is 200 nm so it is large enough to remove the finite size effect. The quantum dot is positioned in the center of flake. We use an open source code package for numerical tight-binding calculations: *Pybinding* (*14*). With the help of fast implementation of kernel polynomial method (*15-16*) in this package, we can quickly obtain the LDOS in an appropriate energy broadening Γ in this about 4 million carbon atoms system and compare them with our experiment results.

We emphasis that the Fermi velocity $v_F \approx 1.26\times10^6\,\mathrm{m/s}$ and $t_{ij} = -3.9eV$ for the GQD (β = 2.4, r_0 = 9 nm, corresponding to the numerical calculation in Fig. 2b, Fig. 3, Fig. 4, Fig. S6b, Fig. S10a, Fig. S12), based on the measured results experimentally. To present better effect, the different energy broadening Γ are used to calculate in different cases. We employ Γ = 0.011 eV to the GQD (β = 2, r_0 = 6.5 nm), Γ = 0.009 eV to the GQD (β = 2.4, r_0 = 9 nm), Γ = 0.02 eV to the GQD (β = 4.3, r_0 = 4 nm). Specially, we emphasis that Γ = 0.003 eV for the map of LDOS as a function of the square root of the magnetic field $\sqrt{B}$ for the GQD (β = 2.4, r_0 = 9 nm) and as a function of β for B = 0 to determine the position of the energy states precisely (corresponding to the numerical calculation in Fig. 4 and Fig. S9).

8. The d*I*/d*V* spectra and simulated LDOS at different GQDs

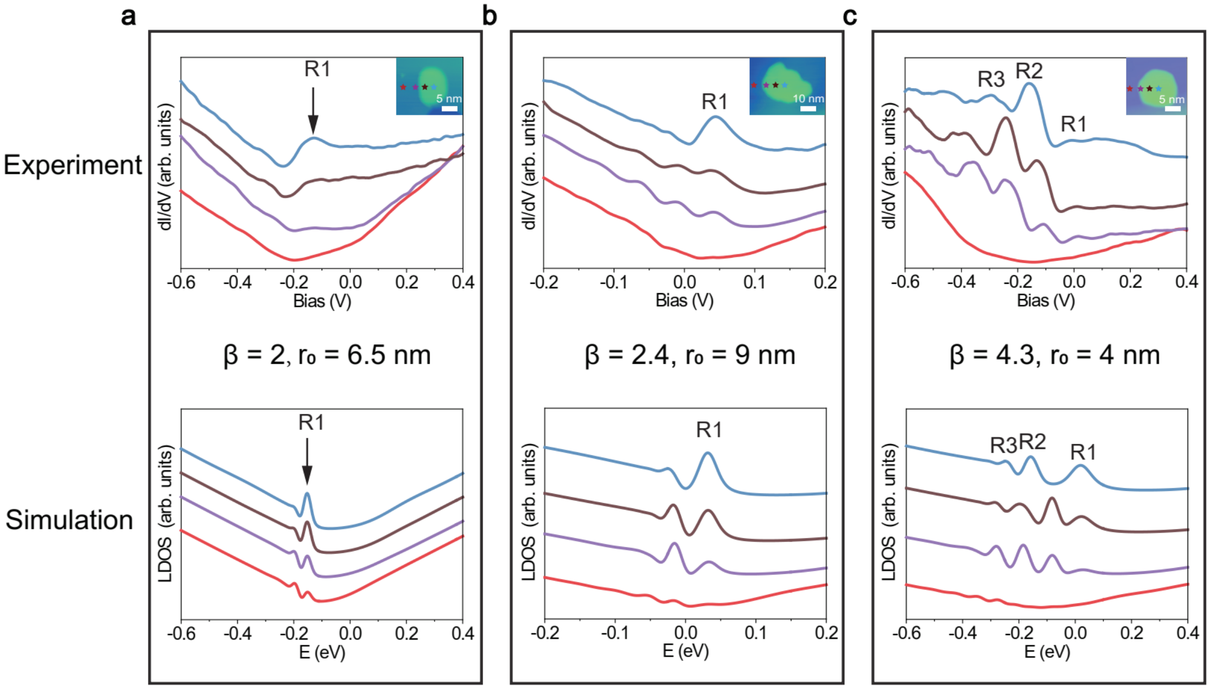

Fig. S6. The d*I*/d*V* spectra (Top panels) and simulated LDOS (Bottom panels) taken at different locations inside and outside different GQDs. **a**, $\beta = 2$, $r_0 = 6.5$ nm. **b**, $\beta = 2.4$, $r_0 = 9$ nm. **c**, $\beta = 4.3$, $r_0 = 4$ nm. Insets of **a** to **c**: the STM images of the GQDs. The spectra with different colors are measured at the positions with the same colored pentagram in the insets. At a smaller β ($\beta = 2$, $r_0 = 6.5$ nm), only one resonance peak (R1) can be observed. With increasing the β, the characteristic of WGMs confinement becomes more pronounced: they display a series of equally spaced resonance peaks at the edge of GQDs. In the case of a larger β ($\beta = 4.3$, $r_0 = 4$ nm), in addition to the quasibound states dominated by WGM confinement at the edge of the GQD, there are three unequally spaced resonance peaks (R1~R3) located at the center.

9. Spatial distribution of ACSs in the GQD

We plot the radially d^2I/dV^2 spectroscopic maps (Fig. S7a) of the GQD with $\beta = 4.3$ (Fig. 1b) to accentuate the striking ACSs features. After differentiation, we can clearly see other two peaks in the center of the GQD. The three unequally spaced resonance peaks are identified as quasibound states due to atomic collapse resonance. For convenience, the two quasibound states are marked as N1' and N1''. As shown in Fig. S7b, the corresponding d^2I/dV^2 spatial maps display strong intensity near the center of the GQD, which is consistent with ACS (*9-10*). To further analyze the properties of these quasibound states (marked by N1~N4, N1' and N1'' in Fig. S7a), the LDOS spatial distribution of the six states in the GQD has been calculated (Fig. S8). The LDOS of quasibound states corresponding to purple hollow dots in Fig. S7a (N1, N1', N1'') gather in the central area. While the LDOS of quasibound states corresponding to the three black solid dots in Fig. S7a show a typical WGM ring-like spatial distribution. These evident traits demonstrate that ACSs and WGMs coexist in the GQD.

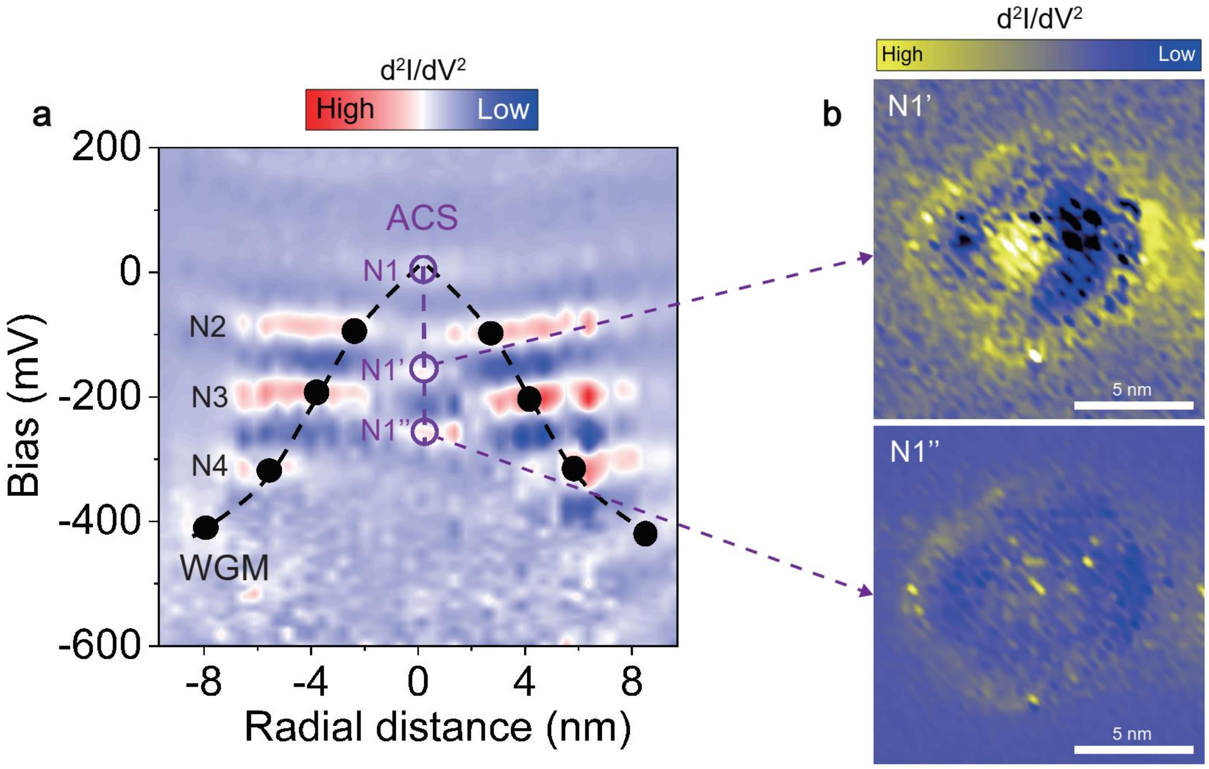

Fig. S7. Spatial distribution of the ACSs in the GQD. **a**, The radially d^2I/dV^2 spectroscopic maps of the GQD (Fig. 1**b**). The quasibound states dominated by WGMs confinement (N2, N3, N4) are indicated by black solid dots, and quasibound states due to the ACSs are indicated by purple hollow dots (N1, N1', N1''). **b**, The corresponding d^2I/dV^2 spatial maps of N1' and N1''. The quasibound states marked by N1' and N1'' display strong intensity near the center of the GQD, which is consistent with **a**.

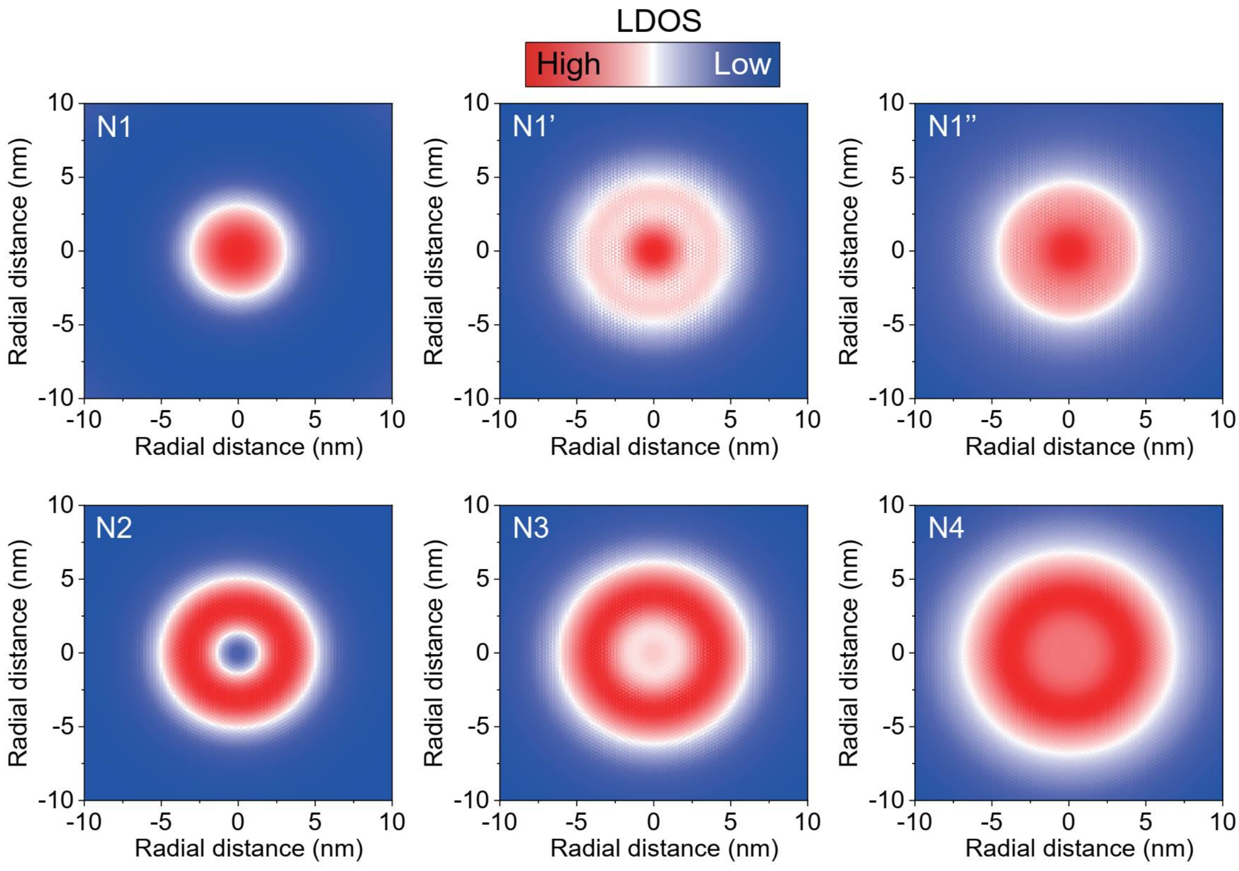

Fig. S8. Simulated maps of LDOS spatial distribution for quasibound states in the GQD ($\beta = 4.3$, $r_0 = 4$ nm), where the N1~N4 states marked in Fig. 1**e** and N1'~N1'' states shown in Fig. S7. N1~N1'' are quasibound states located at the center (corresponding to the purple hollow dots in

Fig. S7a). N2~N4 are quasibound states located at the edge (corresponding to the black solid dots in Fig. S7**a**). Parameters in the calculation: $\varepsilon_i^A = \varepsilon_i^B = -0.32eV$, $t_{ij} = -2.8eV$.

10. Simulated LDOS of quasibound states in the GQD (r_0 = 9 nm) as a function of β and energy

To affirm the existence of ACSs, we further analyze the LDOS for the GQD (r_0 = 9 nm, which corresponds to STM image inset of Fig. S6b) by tuning β at the edge (r = 9 nm) and center (r = 0 nm) of the quantum dot respectively in the absence of magnetic field (Fig. S9a). Fig. S8b shows the cut-off LDOS of Fig. S8a at β = 9 for r = 9 nm and r = 0 nm respectively. We extract the energy positions of peaks in Fig. S8b, and then do a fitting analysis as a function of n (Fig. S9c), where n is the quasibound states index. The energy levels of quasibound states at the edge follows a linear function with a slope around -0.076. While the energy levels of quasibound states at the center follows an exponential function $0.861e^{-0.343n} - 0.042$ approximately. They are well consistent with the theoretical predictions $\frac{\hbar v_f}{R_{eff}} \approx 0.076\left(R_{eff} \approx 11nm\right)$ for WGMs' energy spacing and $\frac{\hbar v_f \beta}{r_0} e^{-\frac{\pi n}{\sqrt{\beta^2 - \left(\frac{1}{2}\right)^2}}}$ -0.040 $\approx 0.831e^{-0.350n} - 0.040$ for ACSs' energy levels (*8*). Thus these data strongly indicate that ACSs appear in the center and, simultaneously, the WGMs confinement appear in the edge of the GQD.

As shown in Fig. S10, we also calculated space-energy maps of the LDOS of the GQD (r_0 = 9 nm) for three different β. The number of quasi-bound states appearing in the center and edge of GQD increases as β climbs. The energy of states in the center shows apparent characteristics of geometric series, which is the representative feature of ACS. While the LDOS at the boundary position show equally spaced peaks, indicating that they are WGM. Although the first peak at the

center of the GQD also exists in the WGM system. We still consider here that it belongs to the ACS. The reason is as follows. In the WGM system, only state with higher orbital angular momentum can be confined well. Since These states of higher orbital angular momentum correspond to an oblique incidence. Klein tunneling gives a nearly perfect reflection for oblique incidence and yields excellent confinement. These quasi-bound states form ring-shaped WGM resonances. While for states with low angular momentum, because of their nearly normal incidence, Klein tunneling brings a closely perfect transmission. The potential well has a weak confinement effect on these states. But for our system, due to the confinement of the Coulomb field, the state of lower orbital angular momentum collapse into ACS and are well localized in the center of the quantum dot. For example, in Fig. 2c of the main text, a series of centered peaks correspond to low angular momentum (e.g. $m = 0$), are the geometric peaks for ACS. The sided peaks for WGM correspond to other high angular momentum. The energy equidistance between these peaks is related to the difference of their angular quantum numbers just by one.

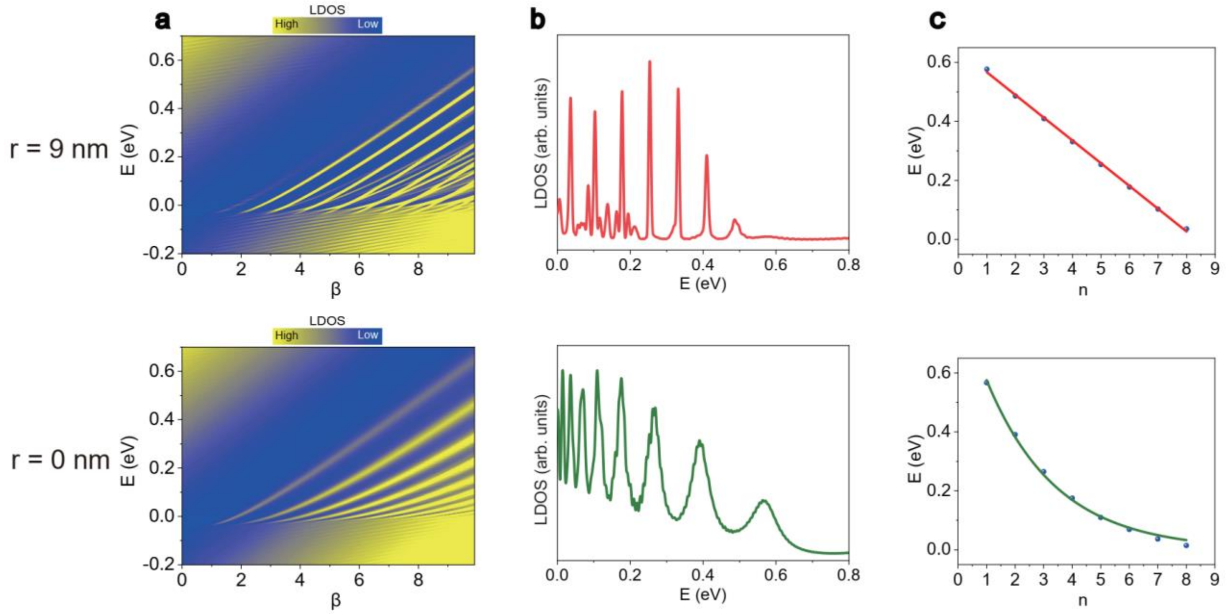

Fig. S9. Simulated maps of LDOS of quasibound states in the GQD ($r_0 = 9$ nm) as a function of β and energy. **a**, LDOS maps of quasibound states located at the edge ($r = 9$ nm) and the center ($r = 0$ nm) of the GQD. The red dots indicate experimental results at $\beta = 2.4$ (the full width at half maximum of the peaks in experiment was used as the error bar), which is well consistent with the simulated results. **b**, Cut-off of LDOS map for **a** at $\beta = 9$. **c**, The fitting of the peaks extracted from **c**. The energy levels of quasibound states at $r = 0$ nm follow a linear function with a slope around -0.076. At $r = 9$ nm, the energy levels of quasibound states display an exponential function $0.861e^{-0.343n} - 0.042$ approximately, where n is the quasibound states index. Parameters in the similation: $\varepsilon_i^A = \varepsilon_i^B = -0.04$, $t_{ij} = -3.9eV$.

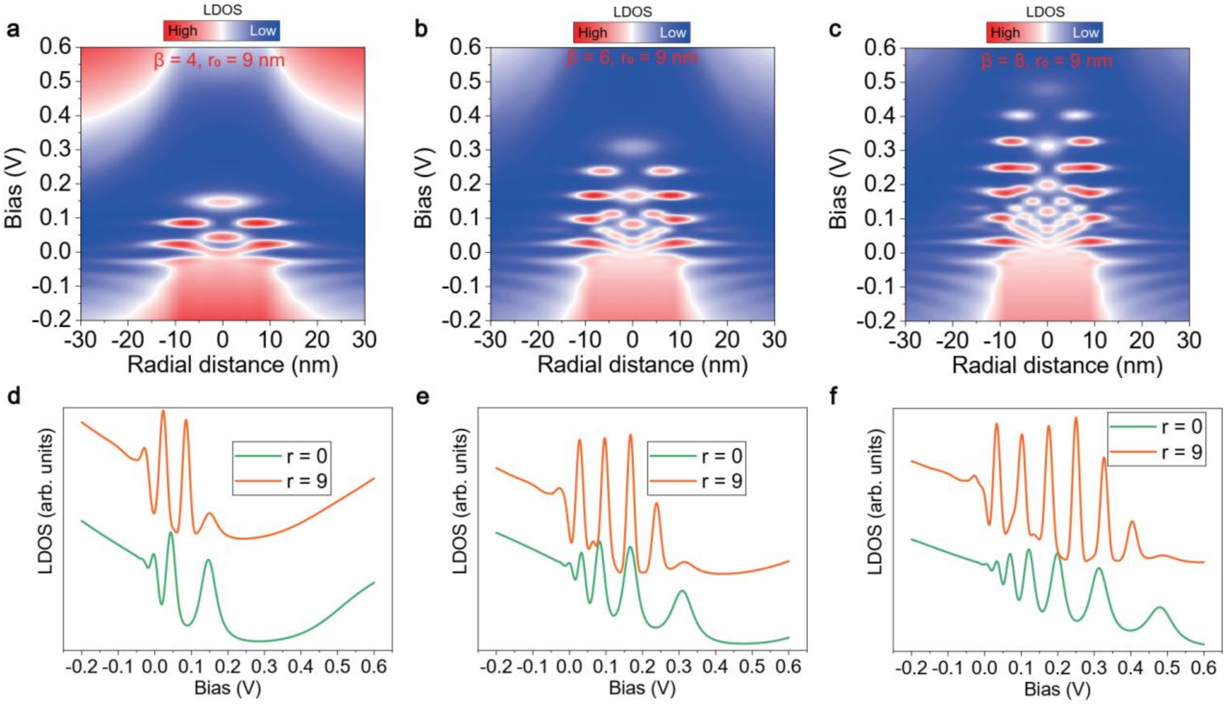

Fig. S10. The calculated space-energy maps of the LDOS of the GQD (r_0 = 9 nm) with different β. **a**, $\beta = 4$. **b**, $\beta = 6$. **c**, $\beta = 8$. **d**, Cut-off of LDOS map for **a** at $r = 0$ and 9 nm. **e**, Cut-off of LDOS map for **b** at $r = 0$ and 9 nm. **f**, Cut-off of LDOS map for **c** at $r = 0$ and 9 nm.

11. Screened electrostatic potential and asymmetry of electron and hole branches

In the presence of a finite magnetic field **B**, normal to the graphene plane, particularly pronounced Landau quantization can be observed (Fig. S10a). According to Equation (1), we plot the measured peak energies of LLs as a function of the magnetic field and level index $\mathrm{sgn}(N)\times\sqrt{|N|B}$, which show $v_F \approx 1.26\times10^6\,\mathrm{m/s}$ and $1.06\times10^6\,\mathrm{m/s}$ to electron and hole respectively. The asymmetry is caused by the Dirac point slightly doped to +45 meV (*17-18*). Near the center of GQD (~30 nm), the peak of 0 LL exhibits significant shifting upwards toward positive energies, which indicates a repulsive potential produced by the GQD (Fig. S10a). We also calculated LDOS map over large distances to the GQD ($\beta = 2.4$, r_0 = 9 nm), which show similar result to experiment. However, there is an obvious difference between calculation and experiment. The electrostatic potential has been affected the Landau level up to the farthest distance in calculation, but only about 30 nm experimentally. This is most likely because the electrostatic potential generated by the GQD would be screened by the charge carriers in graphene.

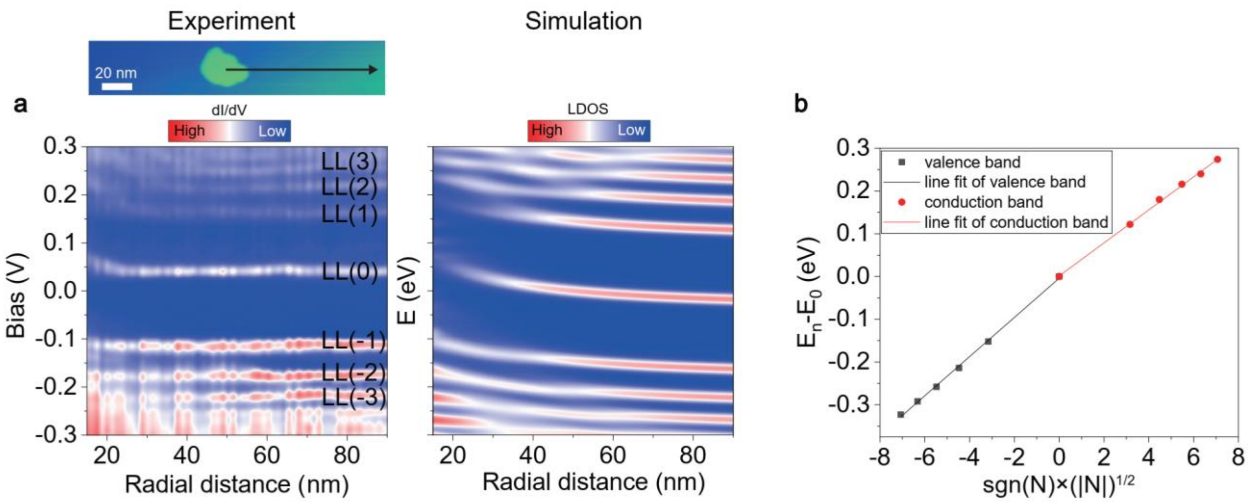

Fig. S11. **a**, dI/dV spectroscopic map (Left) along the black arrow in the top image at B = 10 T, the corresponding calculated LDOS map (Right) for the GQD (β = 2.4). The calculated parameters are the same as simulation in Fig. 3**c**. Pronounced Landau quantization corresponding to the graphene monolayer can be observed. **b**, the measured peak energies of LLs away from the GQD as a function of the magnetic field and level index $\mathrm{sgn}(N)\times\sqrt{|N|B}$. The asymmetry of electron ($v_F \approx 1.26\times10^6\,\mathrm{m/s}$) and hole ($1.06\times10^6\,\mathrm{m/s}$) parts can be observed because that the Dirac point is slightly doped to +45 meV.

12. The others radially d*I*/d*V* spectroscopic maps under a series of magnetic fields

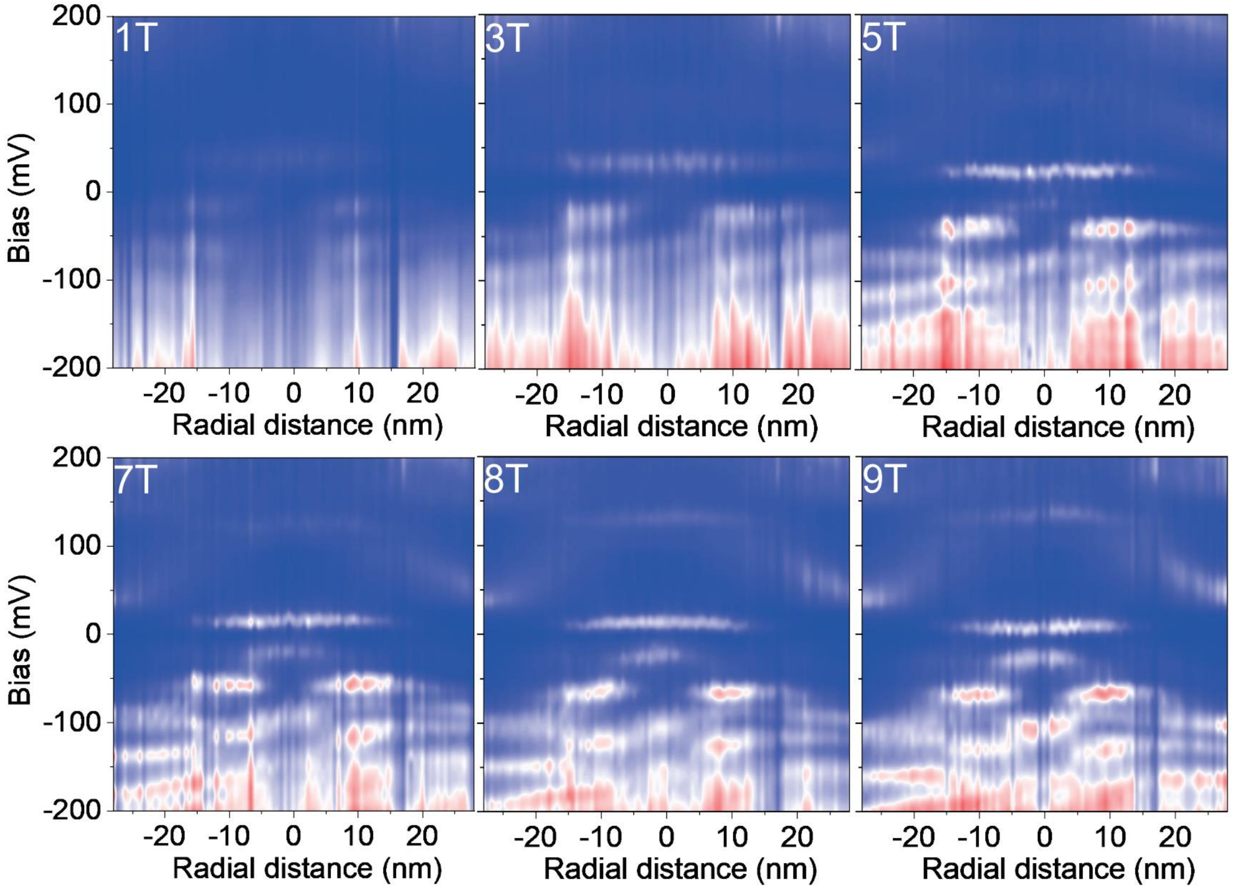

Fig. S12. The radially dI/dV spectroscopic maps on the GQD ($\beta = 2.4$, $r_0 = 9$ nm) in the case of a series of magnetic fields. The corresponding maps with magnetic fields $B = 3.5$ T, 6 T, and 10 T have been shown in Fig. 3**a**- 3**c**. In the case of lower magnetic fields, the map displays almost the characteristics of GQD as that of the zero field (as shown in Fig. 2**b**). With increasing magnetic field, the formation of LLs can be observed in the GQD.

13. The d*I*/d*V* spectra and simulated LDOS at $B = 10$ T

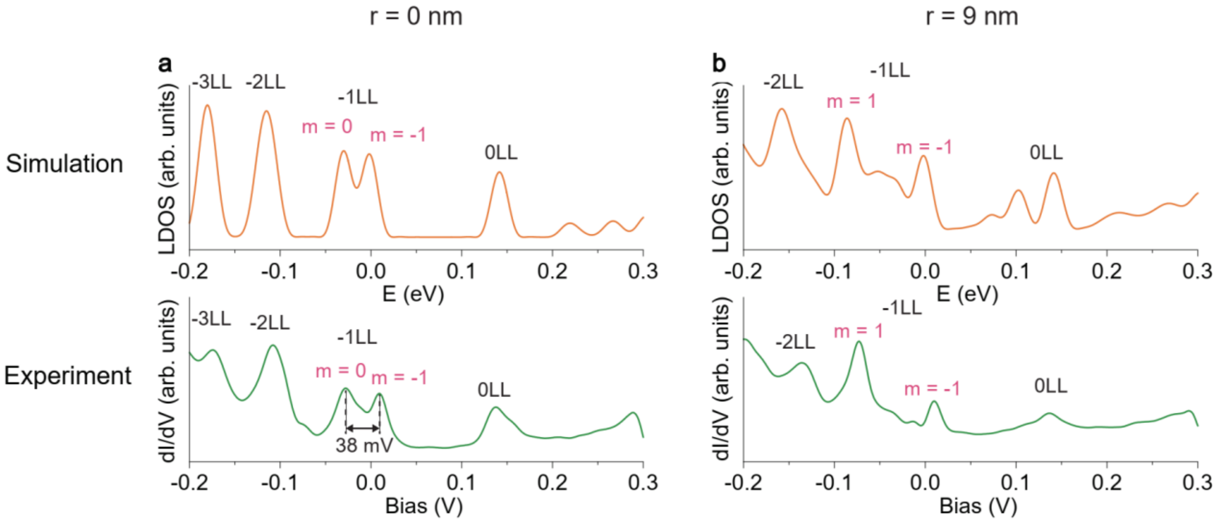

Fig. S13. The dI/dV spectra (Bottom) and simulated LDOS (Top) taken at different locations of the GQD ($\beta = 2.4$, $r_0 = 9$ nm) at $B = 10$ T (i.e. along the vertical line in Fig. 3**c**). **a**, in the center ($r = 0$ nm). **b**, in the edge ($r = 9$ nm). Only electron parts can be observed in the center and edge of GQD. At $r = 0$ nm, -1 LL is split into $m = -1$ and $m = 0$ orbital states with energy gap about 38 meV.

14. The corresponding d*I*/d*V* spectra to experimental data in Fig. 4 in main text and calculated LDOS map with different energy and magnetic field scale

As shown in fig. S14b, in the extremely high magnetic field region ($B > 36$ T), the typical LLs of monolayer graphene have been generated clearly, linear relationship with $\sqrt{B}$. In the case of the extremely high magnetic field, the magnetic length ($l_B = \sqrt{\hbar / eB}$) is much smaller than the cut-off radius (r_0) of the Coulomb potential field, so the influence of the boundary potential field is almost negligible. There is no sign of atomic collapse resonance in this magnetic field region. In the weak magnetic field region ($B < 1$ T), as shown in fig. S14c, a large number of LLs are densely overlapped due to their narrow spacing, and there is a significant enhancement in the

LDOS at the energy position of the collapsed state. This region clearly reflects the characteristics of the ACS. In the high magnetic field region (1 T< B < 36 T), LLs have also appeared. They can be directly connected to the typical LLs in the extremely high magnetic field region, and can also be connected to the ACS of the weak magnetic field region. Among them, we pay attention to the low order LLs (N = 0, -1, -2). They are called as unusual LLs. Their existence is related to the appearance of ACS at zero magnetic field, which are consistent with previous theoretical predictions (*8*). High magnetic field region can also be seen as the transition region from the ACS to the typical LLs.

Now let's analyze the evolution between the typical LLs and ACS in detail from the extremely high magnetic field to the low magnetic field. When the magnetic field is very high ($B \approx$ 140 T), the typical LLs appear as we have mentioned above. As decreasing the magnetic field, the Coulomb potential field begins to affect the LLs, when the magnetic length and cut-off radius can be compared. At this region, the LLs which are originally orbital quantum number degenerate (m > -|N|) begin to split. Different orbital components spread gradually as magnetic field continues to drop. Specially, The LLs significantly bend (N = 0, N = -1, m = 0, -1, N = -2) and show non-linear relationship with $\sqrt{B}$. When the magnetic field is as small about 1T, the distance between different LLs (for example, N= -1, 0, 1, 2, 3...) is hard to be recognized. They converge together, cross over each other and show a signal of ACS (for example, ACS- R1 marked in the Fig. 4). It is worth noting that different orbital components belonging to the same LL may not fall together to the same ACS, such as N = -1, m = -1 and N = -1, m = 0. In addition, as the magnetic field continues to decrease, these LLs still evolve and may move away from the ACS region. For example, after crossing over N = 0, N = -1, m = -1 moves down as magnetic field descends.

The appearance of the unusual LLs reflects the effect brought about by the existence of ACS. Our experiments, complemented by theoretical calculations, explicitly demonstrated the existence of Atomic collapse resonance effect in the presence of high magnetic fields and revealed the close connection between the ACS and the unusual LLs.

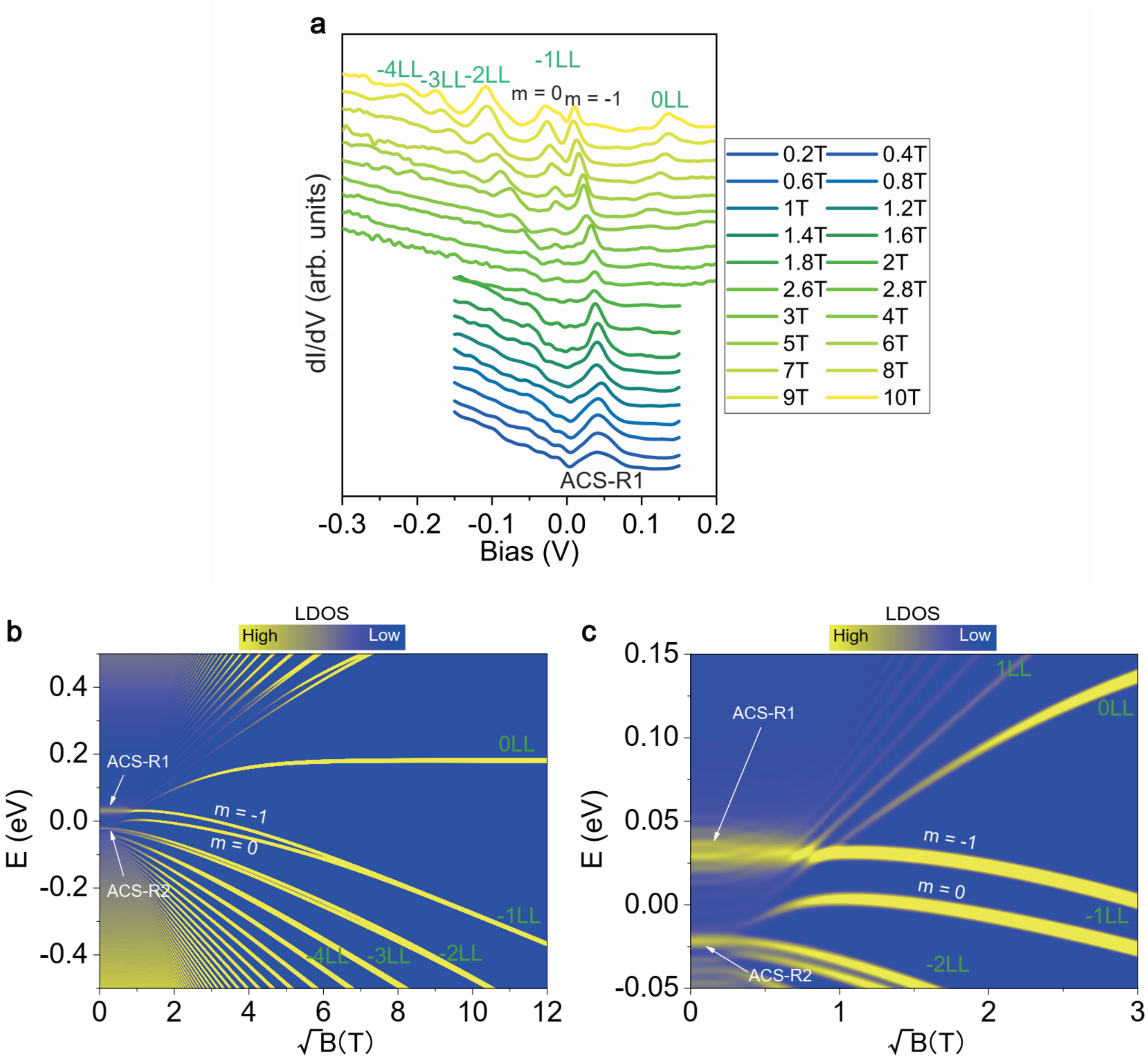

Fig. S14. a, The corresponding dI/dV spectra at the center of the GQD ($\beta = 2.4$, $r_0 = 9$ nm) under different magnetic fields to experimental data in Fig. 4. Only one broad quasibound state (ACS-R1) can be observed in the case of lower magnetic fields, which is well connected to the m

= -1 orbital state of -1 LL at higher magnetic fields. **b** and **c**, The calculated maps of LDOS at the center of the GQD ($\beta = 2.4$, $r_0 = 9$ nm) as a function of the square root of the magnetic field $\sqrt{B}$ for different energy and magnetic field scale. The ACS-R1 and ACS-R2 are two quasi-bound states due to atomic collapse resonance.

15. The calculated LDOS map for Gaussian potential

For Gaussian potential, we firstly fit the zeroth LL in Fig. 3c in the main text and obtained a Gaussian potential $V(r) = 0.2 \times \exp(-r^2/(17.5)^2) + 0.02$. The calculated LDOS maps (Fig. S15a and c) at B = 0 T and B =10 T are close to Fig. 2b and Fig. 3c. As well as, The calculated map of LDOS at the center of the GQD as a function of the square root of the magnetic field $\sqrt{B}$ (Fig. S15d) is also similar the experimental results (Fig. 4 in the main text). This Gaussian potential can be approximate to $V(r) = \dfrac{0.2}{1 + \dfrac{r^2}{(17.5)^2} + \dfrac{r^4}{(17.5)^4}} + 0.02$. The high similarity between Gaussian potential field and Coulomb potential field here leading to the similar results. However, Gaussian potential cannot reproduce all the results. We also fit the Dirac point energy profile of Fig. 2c and obtained a Gaussian potential $V(r) = 0.72 \times \exp(-r^2/(10)^2)\text{-}0.36$. The calculated LDOS map (Fig. S15b) is different from our experiment result in Fig. 2c. The peak of the second ACS is very inconspicuous and the profile of LDOS is not in agreement with the experimental results.

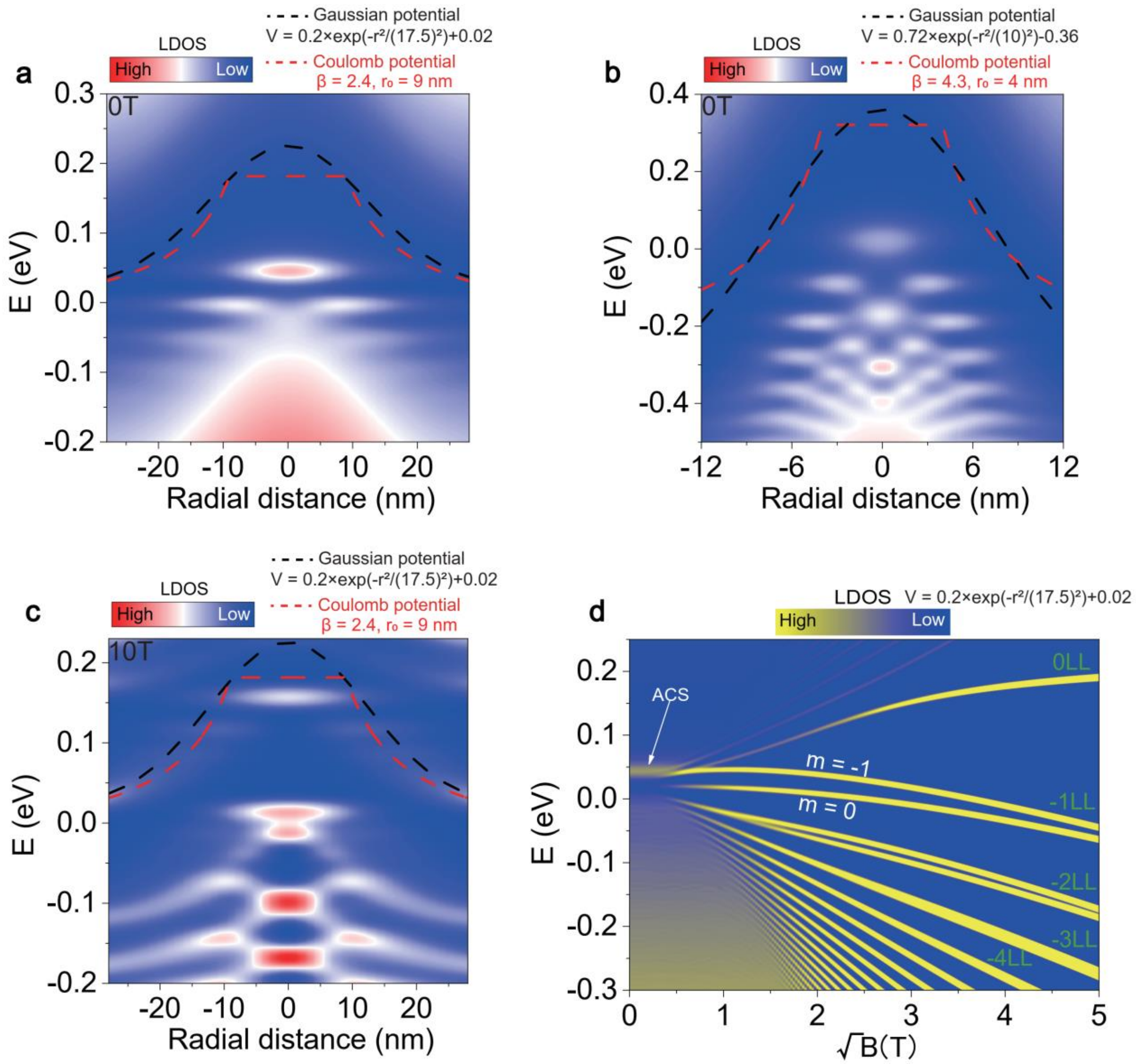

Fig. S15. The calculated the LDOS maps under Gaussian potential. **a**, the calculated space-energy maps of the LDOS of the GQD ($V(r) = 0.2\times\exp(-r^2/(17.5)^2)+0.02$) at B = 0 T. **b**, the calculated space-energy maps of the LDOS of the GQD ($V(r) = 0.72\times\exp(-r^2/(10)^2)$-0.36) at B = 0 T. c, the calculated space-energy maps of the LDOS of the GQD ($V(r) = 0.2\times\exp(-r^2/(17.5)^2)+0.02$) at B = 10 T. The red dotted lines indicate Dirac point energy captured from experiments (**a** and **c**: Coulomb potential, β = 2.4, r_0 = 9 nm; **b**: Coulomb

potential, $\beta = 4.3$, $r_0 = 4$ nm). The black dotted lines indicate Dirac point energy from parabolic potential (**a** and **c**: $V(r) = 0.2 \times \exp(-r^2/(17.5)^2) + 0.02$, **b**: $V(r) = 0.72 \times \exp(-r^2/(10)^2)$-0.36). **d**, The calculated map of LDOS at the center of the GQD ($V(r) = 0.2 \times \exp(-r^2/(17.5)^2) + 0.02$) as a function of the square root of the magnetic field $\sqrt{B}$.

16. The calculated LDOS map for parabolic potential

For parabolic confining potential, as shown in Fig. S16, can in principle generate typical WGM but not ACS, which is consistent with previous study (*19*). When r approaches zero, the decay speed of parabolic confining potential is not fast enough to collapse electrons. In addition, the profile of zeroth LL is hard to fit by r^2. We calculated the LDOS map under potential $V(r) = -0.00024r^2 + 0.02$, as shown in Fig. S16. Although there are similarities between the parabolic potential and the Coulomb potential here. The distribution of calculated LLs under this potential is also obviously difficult to match our experiment.

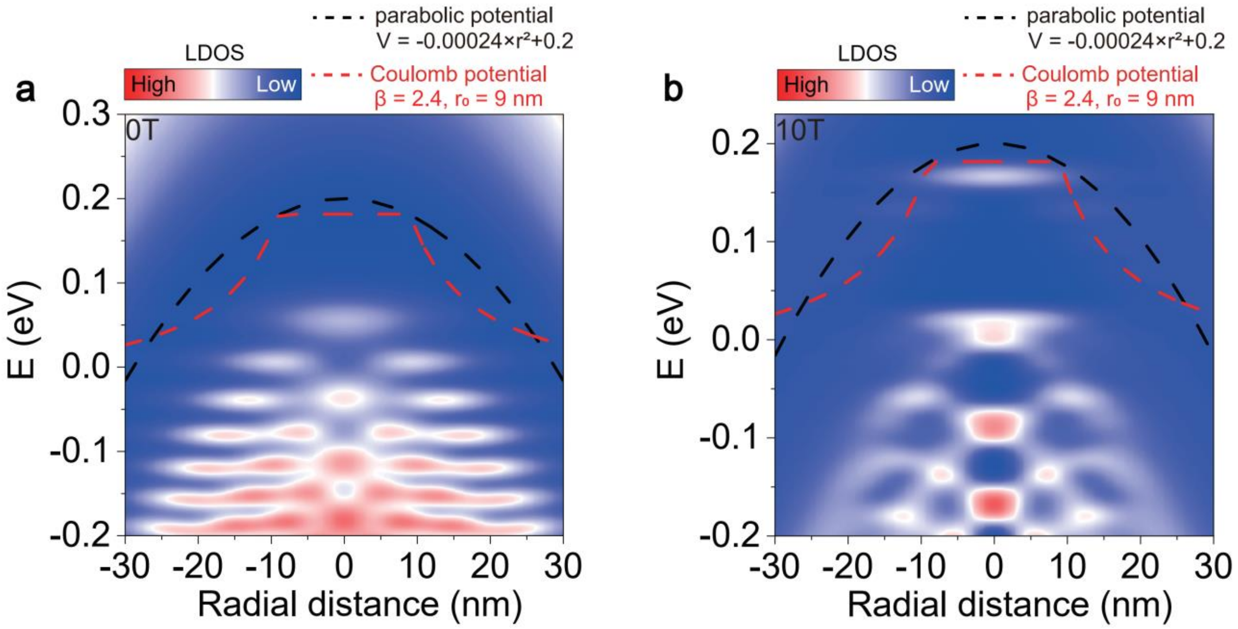

Fig. S16. The calculated the LDOS maps under parabolic potential $V(r) = -0.00024r^2 + 0.02$. a, at $B = 0$ T. b, at $B = 10$ T. The red dotted lines indicate Dirac point energy captured from experiments (Coulomb potential, $\beta = 2.4$, $r_0 = 9$ nm). The black dotted lines indicate Dirac point energy from parabolic potential ($V(r) = -0.00024r^2 + 0.02$).

References

1. Gutiérrez, C., Brown, L., Kim, C.-J., Park, J. & Pasupathy, A. N. Klein tunnelling and electron trapping in nanometre-scale graphene quantum dots. *Nat. Phys.* **12**, 1069-1075 (2016).
2. Bai, K.-K. et al. Generating nanoscale and atomically-sharp p-n junctions in graphene via monolayer-vacancy-island engineering of Cu surface. *Phys. Rev. B* **97**, 045413 (2018).
3. Fu, Z. Q., Bai, K. K., Ren, Y. N., Zhou, J. J. & He, L. Coulomb interaction in quasibound states of graphene quantum dots. *Phys. Rev. B* **101**, 235310 (2020).
4. Zhang, Y. et al. Electronic Structure, Surface Doping, and Optical Response in Epitaxial WSe_2 Thin Films. *Nano Lett.* **16**, 2485-2491 (2016).
5. Addou, R. & Wallace, R. M. Surface Analysis of WSe_2 Crystals: Spatial and Electronic Variability. *ACS Appl. Mater. Interfaces* **8**, 26400-26406 (2016).
6. Kahn, A. Fermi level, work function and vacuum level. *Mater. Horiz.* **3**, 7-10 (2016).
7. Castro Neto, A. H., Peres, N. M. R., Novoselov, K. S. & Geim, A. K. The electronic properties of graphene. *Rev. Mod. Phys.* **81**, 109-162 (2009).
8. Moldovan, D., Masir, M. R. & Peeters, F. M. Magnetic field dependence of the atomic collapse state in graphene. *2D Mater.* **5**, 015017 (2018).
9. Wang, Y. et al. Observing atomic collapse resonances in artificial nuclei on graphene. *Science* **340**, 734-737 (2013).
10. Mao, J. et al. Realization of a tunable artificial atom at a supercritically charged vacancy in

graphene. *Nat. Phys.* **12**, 545-549 (2016).

11. Pereira, V. M., Nilsson, J. & Castro Neto, A. H. Coulomb impurity problem in graphene. *Phys. Rev. Lett.* **99**, 166802 (2007).
12. Shytov, A. V., Katsnelson, M. I. & Levitov, L. S. Vacuum Polarization and Screening of Supercritical Impurities in Graphene. *Phys. Rev. Lett*. **99**, 236801 (2007).
13. Shytov, A. V., Katsnelson, M. I. & Levitov, L. S. Atomic collapse and quasi-rydberg states in graphene. *Phys. Rev. Lett.* **99**, 246802 (2007).
14. Moldovan, D. & Peeters, F. pybinding v0.9.5: a Python package for tight-binding calculations. *Zenodo*. https://doi.org/10.5281/zenodo.4010216 (2020).
15. Weiße, A., Wellein, G. Alvermann, A. & Fehske, H. The kernel polynomial method. *Rev. Mod. Phys.* **78**, 275 (2006).
16. Convaci, L., Peeters, F. M. & Berciu, M. Efficient Numerical Approach to Inhomogeneous Superconductivity: The Chebyshev-Bogoliubov–de Gennes Method. *Phys. Rev. Lett.* **105**, 167006 (2010).
17. Luican, A., Li, G. & Andrei, E. Y. Quantized Landau level spectrum and its density dependence in graphene. *Phys. Rev. B* **83**, 041405(R) (2011).
18. Chae, J. et al. Renormalization of the graphene dispersion velocity determined from scanning tunneling spectroscopy. *Phys. Rev. Lett.* **109**, 116802 (2012).
